# Pharmacogenetic Testing of Children and Adolescents with Mental Health Conditions: Real-World Experiences

**DOI:** 10.3390/ph18081170

**Published:** 2025-08-08

**Authors:** April Kennedy, Sierra Scodellaro, Ruud H. J. Verstegen, Iris Cohn

**Affiliations:** 1 Division of Clinical Pharmacology and Toxicology, The Hospital for Sick Children, Toronto, ON M5G 1X8, Canada; april.kennedy@sickkids.ca (A.K.); sierra.scodellaro@sickkids.ca (S.S.); ruud.verstegen@sickkids.ca (R.H.J.V.); 2 Department of Paediatrics, University of Toronto, Toronto, ON M5G 1X8, Canada; 3 Division of Rheumatology, Department of Paediatrics, The Hospital for Sick Children, Toronto, ON M5G 1X8, Canada

**Keywords:** pharmacogenetic, adverse drug reaction, inefficacy, antidepressants, antipsychotics, pediatric

## Abstract

**Background/Objectives:** Medication discontinuation attributable to adverse drug reactions (ADRs) and/or inefficacy remains a concern of psychotropic medications among children and adolescents. Pharmacogenetic (PGx) testing has been proposed to individualize treatment, although its utility remains uncertain. We retrospectively evaluated whether PGx testing of two key metabolism genes (i.e., *CYP2C19* and *CYP2D6*) explains reported episodes of ADRs and treatment inefficacy experienced by children and adolescents with diverse mental health conditions. **Methods:** PGx testing of *CYP2C19* and *CYP2D6* was conducted for 100 participants before, during, or after the use of psychotropic medication(s) that have clinical practice guidelines supporting PGx-guided dosing. The theoretical impact on medication dosing was reviewed in the context of clinical guidelines. We then evaluated whether the PGx-inferred metabolizer phenotype was consistent with reported ADR and/or treatment inefficacy. **Results:** If PGx testing had been performed before the start of treatment, 43% (35/82) of participants would have been recommended dose adjustments or alternative therapy of at least one medication. PGx test results corroborated 8% (6/76) of ADR events and 3% (2/61) of inefficacies. However, no single participant had all prior reported ADRs or inefficacies explained by the results of *CYP2C19* nor *CYP2D6* testing. **Conclusions:** Reactive testing of *CYP2C19* and *CYP2D6* provided limited insight into isolated incidents of psychotropic medication intolerance in this population. No individual’s PGx test results explained all episodes of ADR or suboptimal response. Variation in drug metabolism genes alone does not provide an explanation for multiple episodes of inefficacy or adverse reaction. In the setting of child and adolescent psychiatry, PGx testing is best suited for preemptive use to complement clinical decision making.

## 1. Introduction

In recent years, psychotropic medications, particularly antipsychotics and antidepressants, have been increasingly prescribed to children and adolescents, with a spike during the coronavirus disease 2019 pandemic [1,2]. Despite the increased use, antidepressant and antipsychotic medications are often discontinued due to inefficacy and/or adverse drug reactions (ADRs) [3,4,5,6]. In pediatric cohorts, antidepressant inefficacy has been observed in 30–60% of participants [7,8,9] and ADRs have been attributable to 2–26% of antidepressant and/or antipsychotic discontinuation [3,10].

Pharmacogenetic (PGx) testing has the potential to increase the clinical response and safety of psychotropic medication [11,12,13,14]. Professional organizations such as the Clinical Pharmacogenetics Implementation Consortium (CPIC) and the Dutch Pharmacogenetics Working Group (DPWG), as well as regulatory agencies such as the U.S. Federal Drug Agency and Health Canada-Santé Canada provide recommendations for modified psychotropic dosing or consideration of alternative therapy based on PGx variation [15,16]. The recommendations in these clinical guidelines are derived from evidence supporting the role of variations in genes encoding pharmacokinetic enzymes, such as CYP2C19 and CYP2D6, on drug metabolism. Clinical PGx guidelines based on these genes are available for certain selective serotonin reuptake inhibitors (SSRIs), serotonin–norepinephrine reuptake inhibitors (SNRIs), atypical antipsychotics, and tricyclic antidepressants [15,16,17,18]. However, the influence of variations in pharmacodynamic genes, *SLC6A4* (a serotonin receptor) and *HTR2A* (a serotonin receptor), on response to psychotropic medications has been assessed by CPIC, which determined that there is insufficient evidence to inform dosing decisions based on variation in these genes at this time [15].

In both adult and pediatric populations, preemptive PGx-guided psychotropic medication dosing has been shown to yield neutral or improved clinical and economic outcomes compared to standard treatment [11,13,14,19,20,21,22]. While many institutions have already implemented a preemptive PGx testing model [23,24,25], broader uptake in the setting of mental health is slow, and more often, testing tends to be conducted reactively to explain previously experienced ADRs or treatment inefficacy [26]. Studies exploring reactive testing have shown inconsistent correlation between PGx-predicted medication metabolism and psychotropic medication response, suggesting that variation in metabolism genes alone is insufficient to explain prior inefficacy and/or ADRs [27,28].

Psychiatric and pediatric consortia have conflicting opinions on the use of reactive versus preemptive PGx testing. For instance, the International Society of Psychiatric Genetics stated that current evidence supporting widespread adoption of PGx testing for psychotropic medications is inconclusive, but endorses using existing PGx test results, such as *CYP2C19* and *CYP2D6* testing, to inform medication decisions in patients who have experienced prior ADR or inefficacy [29]. The American Academy of Child and Adolescent Psychiatry stated that PGx testing should not be used to choose psychotropic medications, citing limited supporting evidence, lack of pediatric study groups in clinical trials, and confounding pharmacodynamic factors [30]. The Canadian Paediatric Society noted that PGx variation infrequently explains prior multi-medication treatment failure, emphasizing limited understanding of the role of pharmacodynamic and other pathways in drug response [31]. Collectively, these recommendations emphasize the need for additional evidence of clinical utility before widespread adoption of PGx testing in pediatric mental healthcare.

Psychotropic medications are increasingly used in pediatric populations, but they are associated with high rates of inefficacy and side effects. PGx testing has been proposed as a way to improve treatment safety and efficacy; however, in clinical practice it is often reserved for children who have failed multiple treatments. While many factors are known to affect responses to psychotropic medications, for this study, we explored whether reactive PGx testing of the *CYP2D6* and *CYP2C19* metabolism genes can offer insight into the causes of treatment failure or ADRs in a cohort of pediatric patients with diverse mental health conditions.

## 2. Results

### 2.1. Cohort Characteristics

A total of 100 participants were included in this study (41% female, mean age ~13 years). Referring provider specialties varied, with the most frequent being pediatricians and child and adolescent psychiatrists, comprising 63% (33/52) of all providers. Participants were diagnosed with diverse mental health conditions, with the most frequent being attention-deficit/hyperactivity disorder, anxiety disorder, autism spectrum disorder, and major depressive disorder. All but one participant were diagnosed with at least one of the aforementioned most frequent disorders, with 39% of participants having two of these diagnoses. All diagnoses are described in Table 1. The total diagnoses collectively exceed 100% as many participants were diagnosed with two or more concurrent mental health conditions.

Psychotropic medications prescribed to participants belonged to several drug classes including SSRIs, SNRIs, tricyclic antidepressants, atypical antipsychotics, central nervous system stimulants, and centrally acting alpha2A-adrenergic receptor agonists. Examination of medication history prior to, and at the time of testing, revealed participants had trialed a median of four (range 0–12) psychotropic medications and one (range 0–6) PGx-guided psychotropic medication. Among the PGx-guided medications, SSRIs were the most frequently prescribed drug class, with sertraline being the most commonly used medication within the cohort (Table 1). Of the medications that did not have clinical PGx guidelines at the time of review, central nervous system stimulants were the most commonly used class, with methylphenidate being the commonly used medication (55% of participants). A detailed breakdown of all medications used in the cohort that did not have clinical PGx practice guidelines available at the time of review is provided in Supplementary Table S1.

### 2.2. CYP2C19 and CYP2D6 Metabolizer Phenotype Frequencies

**Figure 1:**
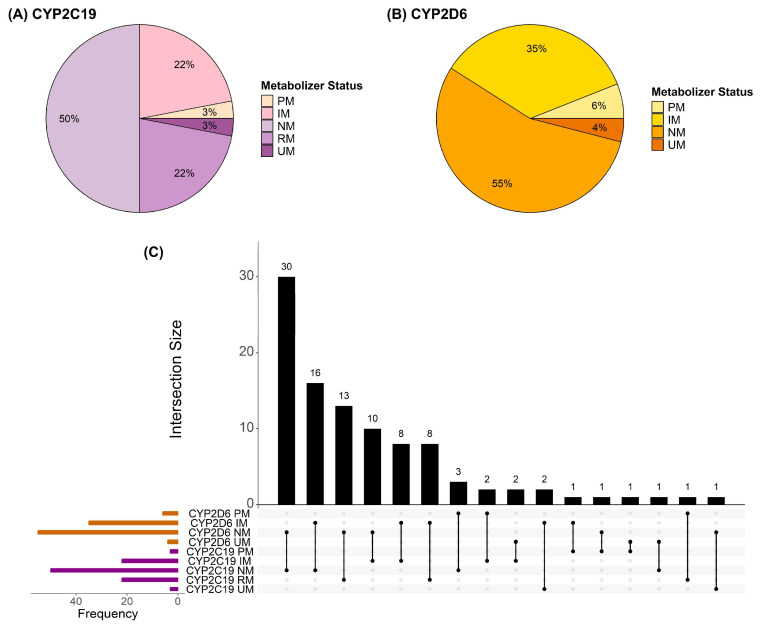
Frequency of metabolizer phenotypes across the cohort (N = 100). (**A**) CYP2C19 metabolizer phenotype frequencies. (**B**) CYP2D6 metabolizer phenotype frequencies. (**C**) UpSet plot depicting the combination of metabolizer phenotypes for both enzymes. The intersection size reflects the number of participants with a specific combination. PM, poor metabolizer. IM, intermediate metabolizer. NM, normal metabolizer. RM, rapid metabolizer. UM, ultrarapid metabolizer.

Based on identified genotypes, the metabolizer phenotypes observed in the study cohort were summarized for the CYP2C19 and CYP2D6 enzymes. The CYP2C19 metabolizer phenotypes followed a normal distribution in the cohort, with 50% of participants having an altered (non-normal) metabolizer phenotype (Figure 1A). The distribution of CYP2D6 metabolizer phenotypes was comparable to previously characterized global distributions [32], with 45% of participants having an altered (non-normal) metabolizer phenotype (Figure 1B). The most common metabolizer phenotype combination was CYP2C19 and CYP2D6 normal metabolizers (30% of participants) (Figure 1C). Hence, 70% of participants were found to have an altered metabolizer phenotype in at least one of the enzymes of interest, which could have influenced the dosing of PGx-guided medications. Of note, the frequencies of *CYP2C19* and *CYP2D6* diplotypes detected in the cohort are documented in Supplementary Table S2.

### 2.3. Theoretical Impact on Dosing

**Figure 2:**
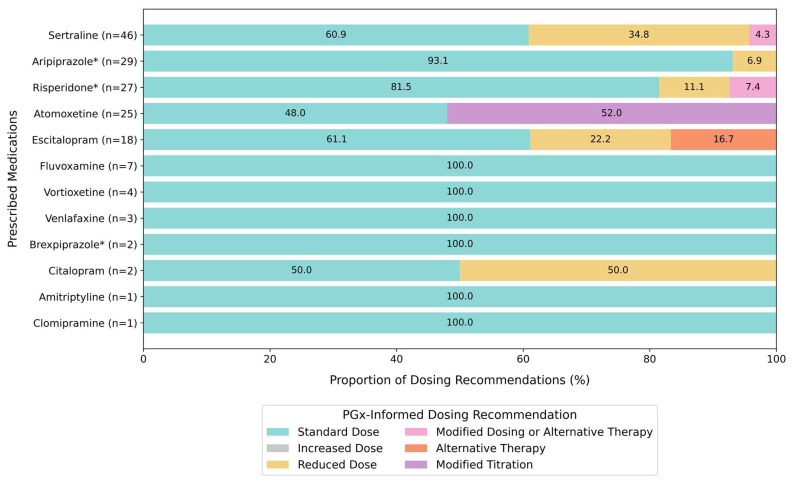
Pharmacogenetic (PGx)-based dosing recommendations for psychotropic medications that have been tried by participants either before or at the time of PGx testing (n = 82 participants). Many participants tried more than one psychotropic medication with clinical PGx guidelines. Sample sizes in brackets indicate the number of participants who had tried each medication. Dosing recommendations were obtained from applicable Clinical Pharmacogenetics Implementation Consortium (CPIC) guidelines for medications without an asterisk and from the Dutch Pharmacogenetics Working Group (DPWG) for medications denoted with an asterisk.

A total of 82 participants had used a PGx-guided medication either before or at the time of testing. To determine if the PGx test results could have impacted the dosing strategy if testing was conducted preemptively, the metabolizer phenotypes for these participants were reviewed in the context of the associated clinical PGx practice guideline (Figure 2). All the participants who had been prescribed fluvoxamine, vortioxetine, venlafaxine, brexpiprazole, amitriptyline, and clomipramine would have been recommended standard dosing based on the participants’ metabolizer phenotypes as per the corresponding clinical PGx guidelines [15,16,17]. A significant portion of the participants who had used sertraline, risperidone, atomoxetine, aripiprazole, escitalopram, and citalopram would potentially have benefited from non-standard dosing based on their metabolizer phenotype. For example, nearly 40% (18/46) of participants who had taken sertraline would have been recommended to consider a dose reduction or alternative therapy. In total, 43% (35/82) of the participants who had used a PGx-guided medication could have potentially benefited from a non-standard dosing recommendation for at least one of the medications they had tried, supporting the utility of preemptive PGx testing to potentially optimize therapy.

### 2.4. Treatment Response

**Figure 3:**
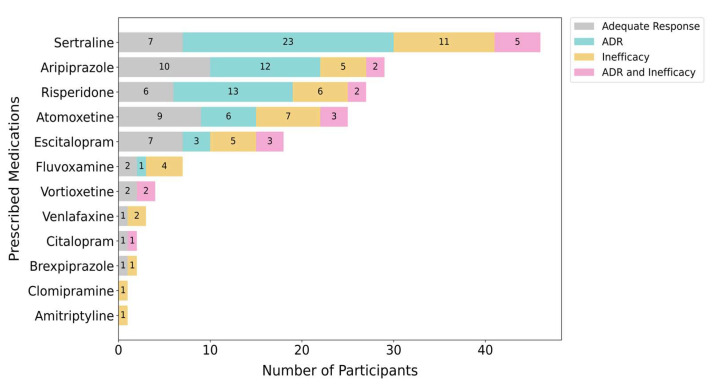
Treatment responses were noted in a subset of participants (n = 69) who had been prescribed a psychotropic medication with a clinical PGx guideline and had reported at least one ADR and/or inefficacy in response to a psychotropic medication with a clinical PGx guideline. Many participants tried more than one psychotropic medication with clinical PGx guidelines. ADR, adverse drug reaction.

Of the 82 participants who had previously tried a PGx-guided medication, 69 had a prior ADR and/or inefficacy as indicated in the referral or electronic health record. The other 31 participants were either referred to the clinic with questions about future PGx-guided medications or had experienced an ADR related to a non PGx-guided medication. Among these 69 participants, 73.9% (51/69) reported at least one ADR, and 43.5% (30/69) had at least one medication inefficacy (Figure 3). Sertraline had the highest proportion of ADRs, occurring in 60.9% (28/46) of participants. However, it is important to note that sertraline was the most frequently prescribed in the cohort; other medications were disproportionately prescribed. The specific ADRs leading to dose reduction or medication discontinuation are described in Supplementary Table S3.

### 2.5. Medication-Centered Analysis: Relationship Between PGx Phenotype and ADRs

**Figure 4:**
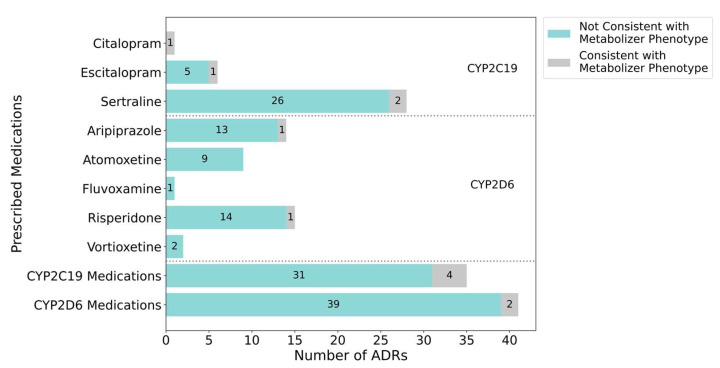
Proportion of ADRs (N = 76) that were consistent with the genetically determined metabolizer phenotype for the primary metabolizing enzyme for each medication. ADRs were documented in the following medications: citalopram, escitalopram, sertraline, aripiprazole, atomoxetine, fluvoxamine, risperidone, and vortioxetine. An ADR was considered consistent with the metabolizer phenotype if the corresponding CPIC or DPWG clinical PGx guideline explicitly mentioned increased risk for side effects/ADRs in association with the participant’s genetically determined metabolizer phenotype and the medication in question. Results that were considered consistent with the metabolizer phenotype are represented by grey bars. Results that were considered inconsistent with the metabolizer phenotype are represented by teal bars. Medications metabolized by CYP2C19 appear in the first segment and medications metabolized by CYP2D6 appear in the second segment, separated by the dashed lines.

Among the eight medications in which an ADR was documented, citalopram, escitalopram, sertraline, aripiprazole, and risperidone were the only ones for which a subset of participants had a metabolizer phenotype that has been associated with increased risk of side effects as outlined in the corresponding CPIC and DPWG guidelines [15,16] (Figure 4). For most medications, CYP2C19 and CYP2D6 intermediate or poor metabolizer phenotypes were considered consistent with an ADR. For certain medications such as aripiprazole, the DPWG guideline does not associate the intermediate metabolizer phenotype with increased risk of side effects. Therefore, such cases were not considered as having a phenotype linked to ADR in our analysis. These scenarios coupled with the participants who were identified as normal, rapid, or ultrarapid metabolizers of the relevant enzyme help explain why the majority of cases of ADRs were not consistent with pharmacogenetic test results. Overall, metabolizer phenotypes aligned with only 7.9% (6/76) of ADRs.

Only one participant reported an ADR for citalopram, which was an oral ulcer and associated oral pain. Dosing information was not available for this participant. A side effect of this nature was not described in the evidence used in the development of the CPIC guideline for citalopram; however, oral sensitivity and burning mouth syndrome have been associated with SSRI use per prior reports [33], and found to be dose-dependent [34]. This participant was a CYP2C19 intermediate metabolizer. The CPIC guideline for citalopram indicates that intermediate metabolizers are at an increased risk of side effects, and thus this outcome was determined to be consistent with the metabolizer phenotype.

As for the six participants who reported an ADR on escitalopram, one participant was determined to be a CYP2C19 intermediate metabolizer, which is consistent with increased risk of side effects per the CPIC guideline. Intermediate metabolizers of CYP2C19 are recommended a slower titration schedule and lower maintenance dose [15]. This participant reported skin irritation/picking, although this side effect is not described in the evidence included in the CPIC guideline. Dosing information was not available for this participant. Four of the other participants with available dosing information experienced an ADR after titration to either 10 mg or 20 mg daily.

One participant out of 14 who had reported an ADR on aripiprazole was determined to be a CYP2D6 poor metabolizer. This metabolizer phenotype is consistent with increased risk of side effects per the DPWG guideline [16]. This participant required a dose reduction due to violent outbursts on 4 mg once daily dosing. Violent outbursts or behavioral changes were not explicitly listed in the evidence synthesized in the DPWG guideline, although certain side effects such as weight gain were found to be associated with the CYP2D6 poor metabolizer phenotype. Per DPWG, a 25–32% aripiprazole dose reduction can be considered in individuals who are determined to be CYP2D6 poor metabolizers [16].

Two out of 28 participants who had received treatment with sertraline were determined to be CYP2C19 poor metabolizers, which is consistent with increased risk for side effects as outlined by the CPIC guideline. Both participants reported altered behaviors that led to discontinuation of sertraline, although information on their dosing regimens was not available. While altered behavior is a known side effect of sertraline, this class of side effect was not explicitly noted in the evidence included in the CPIC guideline [15]. As per CPIC, a lower starting dose, slower titration schedule, and 50% reduction of standard maintenance dose or selection of an alternative agent not predominantly metabolized by CYP2C19 can be considered.

One out of 15 participants receiving risperidone was determined to have a metabolizer phenotype that is consistent with increased risk for ADRs related to this medication. This participant was a CYP2D6 poor metabolizer. The DPWG guideline recommends a 67% dose reduction for CYP2D6 poor metabolizers, and a further dose reduction in the event of side effects [16]. Information on the risperidone dosing regimen was not available for this participant. The ADR in question was hyperprolactinemia, a side effect previously reported in the literature and included as part of the evidence cited by the DPWG in the development of their risperidone guideline [35].

### 2.6. Medication-Centered Analysis: Relationship Between PGx Phenotype and Medication Inefficacy

**Figure 5:**
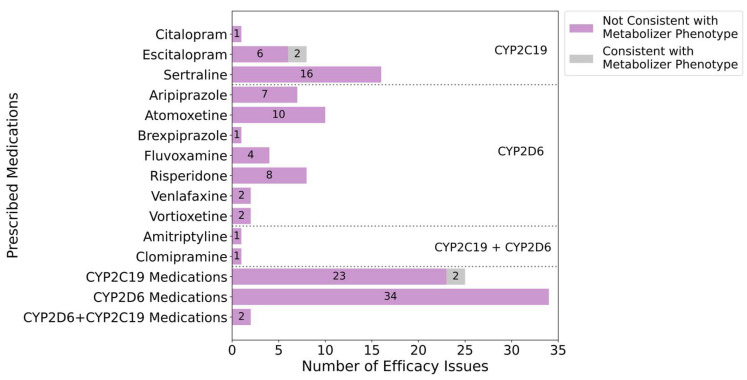
Proportion of medication inefficacy incidents (N = 61) that were consistent with the genetically determined metabolizer phenotype for the primary metabolizing enzyme for each medication. Inefficacy was documented in response to the following medications: citalopram, escitalopram, sertraline, aripiprazole, atomoxetine, brexpiprazole, fluvoxamine, risperidone, venlafaxine, vortioxetine, amitriptyline, and clomipramine. A reported medication inefficacy was considered consistent with the metabolizer phenotype if the corresponding CPIC or DPWG clinical PGx guideline explicitly mentioned either increased risk for therapeutic failure or inefficacy in association with the participant’s genetically determined metabolizer phenotype and the medication in question. Results that were considered consistent with the metabolizer phenotype are represented by grey bars. Results that were considered inconsistent with the metabolizer phenotype are represented by purple bars. Medications metabolized by CYP2C19 appear in the first segment, medications metabolized by CYP2D6 appear in the second segment, and medications metabolized dually by CYP2C19 and CYP2D6 appear in the third segment, separated by the dashed lines.

Of the twelve medications in which inefficacy was noted, escitalopram was the only one where a subset of participants had a metabolizer phenotype that has been associated with an increased risk of therapeutic failure or reduced clinical effect as outlined in the corresponding CPIC guideline [15] (Figure 5). For most medications, CYP2C19 and CYP2D6 rapid (CYP2C19 only) or ultrarapid metabolizer phenotypes were considered consistent with medication inefficacy. However, for certain drugs such as sertraline, CPIC and DPWG guidelines do not associate these phenotypes with reduced effectiveness. In such cases, rapid or ultrarapid metabolizers were not classified as having a phenotype linked to inefficacy in our analysis. This, combined with the number of participants identified as normal, intermediate, or poor metabolizers of the relevant enzyme, helps explain why many cases of treatment inefficacy were not considered consistent with pharmacogenetic test results. Collectively, metabolizer phenotypes were consistent with 3.3% (2/61) of inefficacy across all PGx-guided medications.

Two out of eight participants who experienced escitalopram inefficacy were determined to be rapid metabolizers of CYP2C19. Both participants had reached the maximum daily dose limit for their age (20 mg/day) and thus could potentially have benefited from the CPIC recommendation of an alternative agent not predominantly metabolized by the CYP2C19 enzyme [15].

### 2.7. Participant-Centered Analysis: PGx Testing Utility

**Figure 6:**
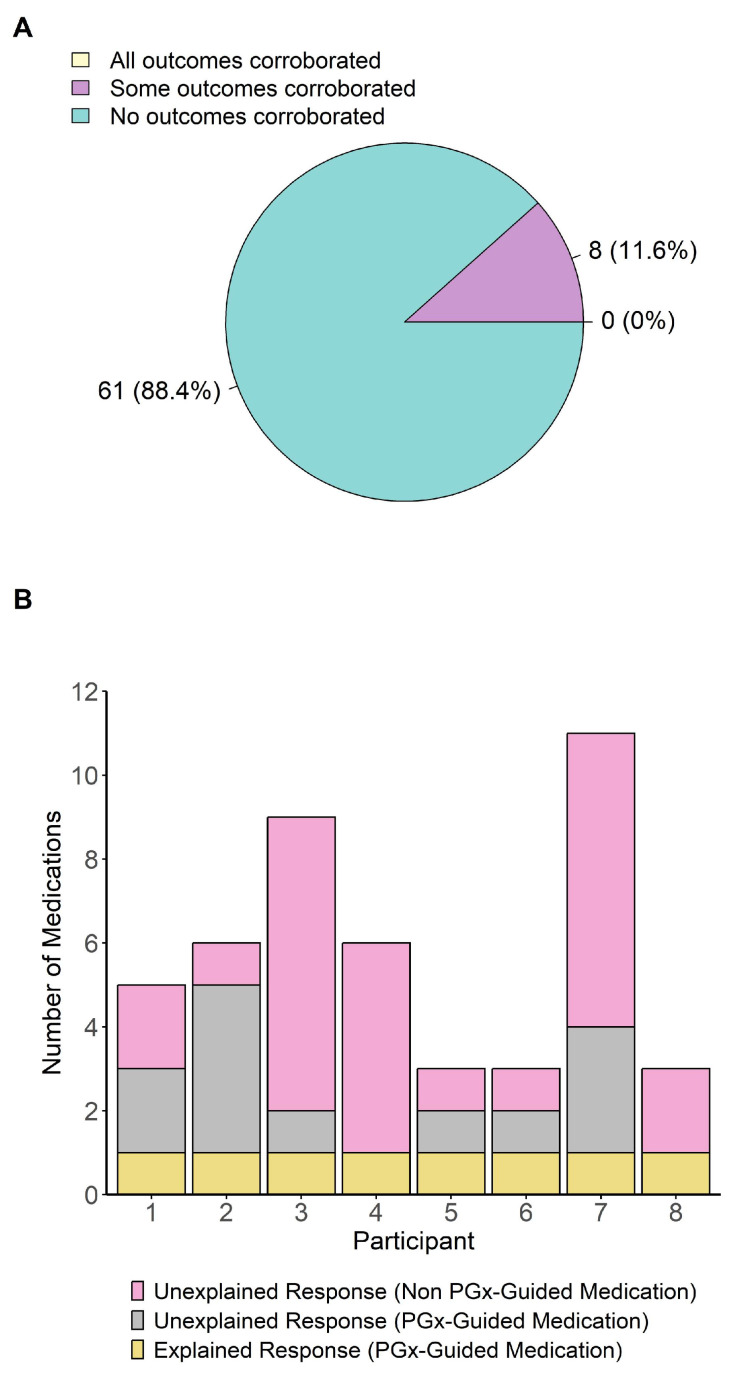
(**A**) Number of participants whose treatment outcome (ADR and/or inefficacy) could be explained by PGx test results. (**B**) Ratio of unexplained versus explained treatment outcomes in the subset of participants who had at least one ADR or inefficacy that could be explained by PGx test results.

While we were able to potentially explain some of the treatment responses by the results of PGx testing (Figure 4 and Figure 5), we wanted to explore how many treatment outcomes of a single patient could be explained by these results. To this purpose, we determined how many reported ADRs and inefficacies were consistent with the metabolizer phenotypes for each individual participant. Of the 69 participants who had an ADR and/or inefficacy in response to a PGx-guided medication, eight (11.6%) were determined to have a metabolizer phenotype that was consistent with treatment response for at least one of the medications they had tried (Figure 6A). When considering PGx-guided medications only, two of these participants’ PGx test results were consistent with all ADRs and/or inefficacy experienced. However, when reviewing all psychotropic medication responses, including medications without a clinical PGx guideline, no single participant’s results could have explained the treatment response for all psychotropic medications they had tried (Figure 6B). Although statistical power was not formally calculated for this analysis, these results suggest that for all participants who presented with more than one episode of ADR and/or inefficacy, PGx testing of *CYP2C19* and *CYP2D6* did not provide a complete explanation for the experienced events.

## 3. Discussion

The rise in psychotropic polypharmacy in children and adolescents in recent years [2] highlights the need to implement strategies to optimize therapy by preventing ADRs and therapeutic failure. While PGx testing of metabolism genes has demonstrated potential in other clinical areas for optimizing and tailoring therapy [36], we here show that in a heterogeneous population of children and adolescents with mental health conditions, reactive PGx testing of *CYP2C19* and *CYP2D6* metabolism genes in the context of ADRs and/or treatment failures provides a limited explanation for these events. We found that only ~8% of ADRs and ~3% of medication inefficacies were consistent with participants’ metabolizer phenotypes; more importantly, there was not a single participant in which PGx test results could explain multiple reported ADRs or inefficacies.

These results suggest that pharmacokinetics play a relatively small role in the development of ADRs or inefficacy, and that other factors should be considered. Diagnosis heterogeneity, psychological factors, social and environmental factors, as well as behavioral and lifestyle factors can all influence the experienced treatment response. From a pharmacologic perspective, variation in pharmacokinetic genes such as *CYP2C19* and *CYP2D6* may explain variability in drug metabolism and resulting drug levels. However, these variations alone do not fully account for interindividual differences in medication response. To illustrate this, prior studies that investigated psychotropic medication outcomes found no significant difference in incidence or severity of side effects in PGx-guided versus standard treatment protocol arms [21]. It can be inferred that the side effects in these participants were not dose-dependent and that other factors, including pharmacodynamics, the mechanisms in which the medication interacts with the body, may be playing a more significant role in response. For example, at the same concentration and CYP2C19 enzyme function, two individuals may respond differently to the same medication, which could in part be attributable to variation in genes that encode for key drug targets such as receptors or components of signaling pathways. Additionally, individuals may exhibit differences in absorption, distribution, and elimination of medications, which can further affect medication response.

The influence of genetic variants on the pharmacodynamic mechanisms that determine medication response to psychotropic agents is not well understood. Several genome-wide association studies have identified candidate genetic variants associated with SSRI and SNRI response, many in transporter and receptor genes, although none were statistically significant at the genome level [37,38,39,40]. These studies have predominately been conducted in adult cohorts of European ancestry and require replication in genetically diverse populations. At this moment, most data are available for *SLC6A4* and *HTR2A* in relation to SSRI response. However, CPIC concluded that the current evidence is mixed and insufficient to support clinical decision making [15]. A recent systematic review described several pharmacodynamic genes (e.g., *ABCB1, BDNF*) with statistically significant association to antidepressant response that were replicated across some, but not all studies evaluated [41]. Continued replication of these findings particularly in diverse cohorts will be necessary to inform future pharmacodynamic-specific PGx dosing guidelines. To this point, our analysis focused on the effect of variations in metabolism genes on psychotropic medication response, as clinical PGx-guided dosing recommendations are limited to these genes [15,16,17,18]. Medications that do not have corresponding clinical PGx guidelines, such as central nervous system stimulants and alpha-2A agonists, tried by many participants, require further characterization of the mechanisms underlying response. There is a significant clinical need to enhance our understanding of the mechanisms behind medication responses in order to explain ADRs and the inefficacy of psychotropic medications.

It is important to recognize the limitations of reactive PGx testing following prior ADRs and/or treatment inefficacy. As previously discussed, other factors are more likely to explain adverse treatment outcomes. As such, standard dose escalation recommendations based solely on metabolism gene variation may not be appropriate for individuals with heightened sensitivity to ADRs, as this could increase their risk of further adverse effects.

While *CYP2C19* and *CYP2D6* genotyping has limited ability to explain ADRs and inefficacy of psychotropic medications, preemptive testing may still offer clinical benefit by optimizing therapy for PGx-guided medications. Our study found that 70% (70/100) of participants were determined to have a non-normal metabolizer phenotype for CYP2C19 and/or CYP2D6. When considering medication use in the cohort and the associated clinical PGx guidelines, 43% (35/82) of participants would have been recommended non-standard dosing or use of an alternative agent for at least one medication they had tried, had this information been available at the time of prescription. Hence, guideline recommendations are most useful for patients who are treatment-naïve. Furthermore, the utility of preemptive PGx testing in the setting of mental health has been demonstrated in larger clinical trials and meta-analyses, whereby participants in PGx-guided dosing arms had improved rates of response and remission compared to the treatment as usual arms [13,14]. In addition, there is evidence supporting the economic benefits of PGx testing in this setting. A recent study conducted in British Columbia, Canada employed a microsimulation model which projected health system savings of CAD 956 million over a 20-year period from PGx-guided care in the setting of major depressive disorder alone [42]. However, the true impact of PGx on the management of children and adolescents with mental health conditions needs to be studied specifically.

The findings presented herein have implications that extend beyond mental healthcare. The CYP2C19 and CYP2D6 enzymes are involved in the metabolism of many medications, with 15–25% of drugs metabolized by CYP2D6 [43]. While we did not review the impact on medications indicated for other clinical uses, it is important to note that participants’ PGx test results described herein may also guide future medication selection and dosing for other indications, thus broadening the clinical actionability and potential impact of preemptive PGx testing in this cohort.

In contrast to the limited clinical utility of reactive PGx testing observed in this study, we noted that children and adolescents, their families, as well as referring providers were positive about the perceived value of pharmacogenetic testing. This sentiment has been observed in pediatric and adult cohorts with mental health conditions whereby a majority of participants expressed positive views toward testing; a subset of participants noted improved outcomes [44,45]. While healthcare providers are keen to incorporate PGx testing into their practice and see the potential benefits for treatment optimization, they acknowledge the need for more education on how to interpret and apply the results. Recurrent concerns include the limited evidence supporting PGx-guided dosing and questions around the role of PGx variation in psychotropic medication response in general. Healthcare providers have also expressed uncertainty with how to best educate and disseminate results to patients [46,47,48,49]. It is critical to enhance healthcare provider understanding of the utility and limitations of PGx testing and the results presented here support that implementation efforts should focus on preemptive rather than reactive testing to help guide pharmacotherapy for the management of mental health conditions in children and adolescents.

The main limitation of this study is that the participants’ medication history was collected retrospectively through a review of their medical records in the electronic health records and/or referral notes, which may have contained inaccuracies. In defining ADR and inefficacy for the purposes of this study, we attempted to identify whether the outcome could be attributed to a single medication, which was limited for the same reason. Future prospective studies would allow us to assess medication history and outcomes more comprehensively.

Another limitation was that we only assessed genetic variations in *CYP2C19* and *CYP2D6*, which have the strongest evidence supporting a role in medication response, specifically metabolism, as reflected in multiple clinical PGx guidelines. However, additional clinical practice guidelines exist. For example, a CPIC guideline for sertraline provides dosing recommendations based on genotyping of *CYP2B6*, and a DPWG guideline for quetiapine provides dosing guidance based on the *CYP3A4* genotype [15,16]. It is possible that the addition of *CYP2B6* could have explained adverse reactions to sertraline for participants in which the *CYP2C19* metabolizer phenotype did not provide answers, although this would only be applicable in the case of CYP2B6 poor metabolizers. *CYP3A4* testing may have provided insight into quetiapine response, although only two participants had experienced an ADR while taking this medication. Future studies could leverage more comprehensive PGx testing including these genes.

Finally, there are other factors that may contribute to medication inefficacy and ADRs that we did not account for due to the limited view of medication and clinical history, particularly for externally referred participants, including drug–drug interactions, comorbid conditions, medication adherence, formulation excipients, and extrinsic factors (i.e., environmental stressors). In determining what constituted an ADR or medication inefficacy, we attempted to identify details on drug dosing and duration, as well as adherence in the provided documentation from the referring provider, although this may not have been complete and reflective of a participant’s true drug exposure.

Additionally, drug–drug interactions, such as the inhibitory effect of certain SSRIs (e.g., fluoxetine, bupropion, and paroxetine) on CYP2D6 enzyme activity, can be a source of discrepancy between the predicted metabolizer phenotype and the actual clinical phenotype, a phenomenon known as phenoconversion [50,51]. We did not quantitatively assess the possibility of phenoconversion given the limited view of medication history, particularly with respect to concomitant medication use. A sensitivity analysis was not conducted to account for the possible influence of these confounding factors, which may underestimate non-genetic causes of treatment response variability. Future work will need to account for the aforementioned variables that are known to influence drug response to better isolate the influence of variation in metabolism genes on psychotropic response.

Future research in this area is anticipated to leverage machine learning approaches to integrate not only variation in pharmacokinetic and pharmacodynamic genes, but other variables such as disease state and environmental influences to better characterize the mechanisms underlying psychotropic medication response [41,52]. In summary, there are many factors that contribute to interindividual differences in medication response beyond variation in pharmacokinetic genes that need to be further studied to optimize psychotropic medication therapy.

## 4. Materials and Methods

### 4.1. Study Cohort

This study involved children and adolescents (age < 18 years) who had PGx testing completed by the division of Clinical Pharmacology & Toxicology at the Hospital for Sick Children (SickKids), Toronto, ON, Canada. between February 2020 and May 2024. Participants were eligible for inclusion if they met the following criteria: (1) diagnosis of a mental health condition, (2) referred for PGx testing regarding the use of psychotropic medications, and (3) at least one of the past, current, or future medications listed in the referral should have published clinical PGx practice guidelines available from either CPIC or DPWG at the time of review (i.e., ‘PGx-guided medication’).

No distinction was made a priori regarding the reason for testing. As a result, this cohort included participants who (1) experienced a prior ADR or inefficacy in response to a PGx-guided medication, or (2) were currently taking a PGx-guided medication at the time of testing, or (3) were PGx-medication-naïve and seeking future prescription guidance.

Cohort characteristics including mental health condition diagnoses, age, sex, and psychotropic medication history were obtained from a retrospective review of referrals and electronic health records. This study was approved by the research ethics board of SickKids and written informed consent was obtained from participants or their substitute decision makers. Special considerations around consent of pediatric study participants were followed in accordance with institutional protocol. Consent forms and consent discussions covered the scope of PGx testing, benefits and risks, anticipated findings, data security and privacy considerations, instructions for consent withdrawal, and relevant resources. Special measures around data governance and security were discussed and reflected in the consent forms. All study data was deidentified by assigning a unique study ID to each participant. More importantly, patient identifiers (e.g., name, full date of birth) were not collected, and in respect to date of birth, only the month and year of birth were collected. The master study log is stored on a separate encrypted server with restricted access. Raw genetic data was stored separately from clinical outcome data.

### 4.2. PGx Analyses

Buccal swabs were collected from participants for PGx testing. Extraction of DNA from buccal swabs and targeted genotyping was performed by Gene by Gene (Houston, TX, USA) using a custom VeriDose^®^ Core panel (Agena Bioscience, San Diego, CA, USA). Furthermore, copy number variation and hybrid alleles of *CYP2D6* were analyzed using the VeriDose^®^ CYP2D6 Copy Number Variation panel on the MassARRAY system. Haplotypes were analyzed using MassARRAY Typer Analyzer (version 5.0.2) and iPLEX ADME PGx Pro (version 3.99.105) software, and PGx test reports were prepared by DNALabs (Toronto, ON, Canada).

The custom Veridose^®^ Core panel interrogates a subset of genetic variants in key pharmacogenes that have an established gene–drug relationship and corresponding published clinical PGx guideline(s). These pre-selected variants affect drug-metabolizing enzyme function or influence drug sensitivity. The variants tested do not have known association with inherited traits or conditions. Hence, the risk of incidental findings was determined to be negligible given the targeted interrogation of pre-selected variants in genes known to impact drug metabolism or sensitivity.

Subsequent analyses focused on *CYP2C19* and *CYP2D6*, which encode primary metabolizing enzymes of PGx-guided medications. Although sertraline dosing is also guided by *CYP2B6*, this gene was added to our custom panel towards the end of the study period, and results were only available for a minority of patients (~10%). For this reason, this gene was excluded from our analyses.

### 4.3. Evaluation of Treatment Response

For participants who were treated with one or more PGx-guided medications, details of treatment response were retrospectively extracted from referral notes, clinical consultation notes, and/or the electronic health record at our institution. Treatment response was categorized as follows: (1) ADR, (2) inefficacy, or (3) ADR and inefficacy. For the purposes of this analysis, an ADR was defined as an undesired effect of a medication that led to a dose reduction or discontinuation. ADRs included in this analysis were determined to be common or plausible reactions in response to the corresponding psychotropic medication in question. Inefficacy was defined as a lack of symptom resolution that led to a dose increase above usual therapeutic dosing or medication discontinuation. The combination of ADR and inefficacy was assigned if the participant had reports of both outcomes during the treatment course for a single medication.

### 4.4. Diplotype to Phenotype Translation: Theoretical Impact on Dosing

Diplotype to metabolizer phenotype translation was performed using CPIC diplotype–phenotype tables [53,54]. Based on the detected diplotypes, participants were categorized either as normal, intermediate, poor, or ultrarapid metabolizers of CYP2D6. For CYP2C19, participants were categorized as either normal, intermediate, poor, rapid, or ultrarapid metabolizers. Distribution of metabolizer phenotypes across the cohort was summarized. We subsequently determined if the metabolizer phenotypes would have led to dosing modification for previous or current PGx-guided medications or selection of an alternative therapy based on clinical PGx practice guidelines, which was defined as actionability of the test results. For example, if a participant was determined to be a CYP2C19 poor metabolizer, the recommendation for sertraline would have been to start with a lower dose, apply a slower titration schedule, and reduce the final target dose by 50% compared to normal metabolizers per the CPIC guideline for CYP2C19 and sertraline [15]. Thus, this finding would be considered actionable had the participant received this information preemptively.

Dosing recommendations from CPIC guidelines were used for sertraline, escitalopram, citalopram, clomipramine, fluvoxamine, vortioxetine, venlafaxine, amitriptyline, and atomoxetine, while recommendations from DPWG were used for brexpiprazole, risperidone, and aripiprazole [15,16,17,18].

### 4.5. PGx Phenotype and Treatment Response: Medication-Centered Analysis

Subsequently, we summarized reported treatment responses within the study cohort. We further evaluated whether a participant’s metabolizer phenotype could have played a role in and/or predicted the treatment response, specifically prior ADRs and/or inefficacy. By evaluating evidence included in clinical PGx practice guidelines, we determined if prior ADRs and/or inefficacy experienced by participants were considered consistent with their metabolizer phenotypes. The impact of enzyme metabolizer phenotype on ADRs and efficacy is medication-specific; therefore, each medication guideline was taken into consideration in isolation. If the CPIC or DPWG guideline explicitly noted that the assigned CYP2C19 or CYP2D6 phenotype has been associated with an increased risk for an ADR for the medication in question, then the participant’s ADR was considered to be consistent with their metabolizer phenotype. For example, if a participant was determined to be a CYP2C19 poor metabolizer and had experienced an ADR on sertraline, this treatment response was considered to be consistent with the metabolizer phenotype, as supported by the CPIC guideline for sertraline [15]. Likewise, if the CYP2C19 or CYP2D6 phenotype has been associated with an increased risk for therapeutic failure for the medication in question, then the medication inefficacy was considered to be consistent with their metabolizer phenotype. To illustrate this, the CPIC guideline for escitalopram/citalopram notes that lower drug plasma concentration attributable to CYP2C19 ultrarapid metabolizer phenotype reduces the clinical effect. In this scenario, if a participant experienced prior citalopram inefficacy, this treatment response was considered to be consistent with their metabolizer phenotype. A summary of the CPIC and DPWG guidelines used to determine whether metabolizer phenotype was consistent with an ADR or inefficacy is included in Supplementary Table S4.

### 4.6. PGx Phenotype and Treatment Response: Participant-Centered Analysis

In contrast to the prior analysis, where we reviewed each ADR and inefficacy per medication, here we determined to what extent PGx testing of *CYP2C19* and *CYP2D6* can explain all instances of psychotropic medication inefficacies and/or ADRs per participant. We evaluated the proportion of each participant’s medication related ADRs and/or inefficacies that were consistent with metabolizer phenotypes, including response to psychotropic medications without clinical PGx guidelines. Inherently, an ADR or inefficacy in relation to a medication that does not have a clinical PGx guideline could not be explained by metabolizer phenotypes, nor could dosing have been modified in theory if testing had been conducted preemptively, but this approach provides a complete view of the utility of PGx testing in this cohort.

## 5. Conclusions

We here demonstrated that although reactive PGx testing of *CYP2C19* and *CYP2D6* can provide limited insight into isolated incidents of psychotropic medication intolerance, it does not account for multiple episodes of inefficacy or adverse reactions experienced across multiple medications. Given the limited explanatory power of testing pharmacokinetic genes in the context of psychotropic medication response, this approach is best positioned as a preemptive tool to guide dosing in treatment-naïve children and adolescents. To improve treatment and outcomes, further research on children and adolescents with mental health conditions should consider the impact of additional genetic and extrinsic factors to better understand the mechanisms underlying psychotropic medication response.

## Figures and Tables

**Table 1:** Cohort characteristics (N = 100).

Characteristic	Result
Age in years: mean [range]	12.7 [6–18]
Sex	
**Female:**	41%
**Male:**	59%
Referral Type	
**Internal ^a^:**	40%
**External ^b^:**	60%
Referring Provider Specialty ^c^: n	*n* = 52
**General pediatrician**	18
**Child and adolescent psychiatrist**	15
**Developmental pediatrician**	4
**Clinical geneticist**	4
**Primary care nurse practitioner**	2
**General practitioner/family physician**	2
**Pediatric anesthesiologist (chronic pain team)**	2
**Other ^d^**	5
Clinical Diagnoses ^e^	
**Attention-deficit/hyperactivity disorder:**	56%
**Generalized anxiety disorder:**	54%
**Autism spectrum disorder:**	39%
**Major depressive disorder:**	28%
**Obsessive–compulsive disorder:**	13%
**Psychosis or psychotic disorder:**	7%
**Oppositional defiant disorder:**	7%
**Social anxiety:**	6%
**Tourette syndrome:**	5%
**Insomnia or sleep disorder:**	5%
**Avoidant-restrictive food intake disorder:**	4%
**Disruptive mood dysregulation disorder:**	2%
**Post-traumatic stress disorder:**	2%
**Bipolar disorder:**	2%
**Other ^f^:**	10%
Pharmacogenetic (PGx)-guided medications tried by cohort ^g^	
***Selective serotonin reuptake inhibitors***	
**Sertraline**	46%
**Escitalopram**	18%
**Fluvoxamine**	7%
**Vortioxetine**	4%
**Citalopram**	2%
***Serotonin–norepinephrine reuptake inhibitors***	
**Atomoxetine**	25%
**Venlafaxine**	3%
***Atypical antipsychotics***	
**Risperidone**	27%
**Aripiprazole**	29%
**Brexpiprazole**	2%
***Tricyclic antidepressants***	
**Amitriptyline**	1%
**Clomipramine**	1%
Average number of PGx-guided psychotropic medications tried by participants: median [range]	1 [0–6]

^a^ Referrals that originated from healthcare providers within the Hospital for Sick Children, Toronto, ON, Canada. ^b^ Referrals that originated from healthcare providers outside of the Hospital for Sick Children, within the province of Ontario. ^c^ A subset of providers referred more than one patient. ^d^ ‘Other’ referring provider specialties included an adolescent medicine physician, a pediatric neurologist, a pediatric endocrinologist, a pediatric immunologist, and a pediatric rheumatologist. ^e^ Participants frequently had multiple concurrent diagnoses and thus percentages across each mental health condition exceed 100%. ^f^ ‘Other’ includes the following conditions that were each observed once: adjustment disorder, pediatric acute-onset neuropsychiatric syndrome, hypochondriasis, schizoaffective disorder, conversion disorder, selective mutism, panic disorder, behavioral disorder, intermittent explosive disorder, and separation anxiety. ^g^ Pharmacogenetic (PGx)-guided medications refer to those that have a clinical dosing guideline from either the Clinical Pharmacogenetics Implementation Consortium (CPIC) or the Dutch Pharmacogenetics Working Group (DPWG). Drug classes are italicized.

## Data Availability

The data presented in this study are available on reasonable request from the corresponding author due to privacy and ethical reasons.

## Supplementary Materials

The following supporting information can be downloaded at https://www.mdpi.com/article/10.3390/ph18081170/s1, Table S1: Cohort (N = 100) use of psychotropic medications that do not have available clinical PGx guidance based on *CYP2C19* or *CYP2D6* genotyping from CPIC or DPWG; Table S2: *CYP2C19* and *CYP2D6* diplotype frequencies observed in the cohort; Table S3: Description of reported adverse drug reactions (ADRs) across the cohort; Table S4: Interpretation of Clinical PGx Guidelines.

## Abbreviations

The following abbreviations are used in this manuscript:

PGx	Pharmacogenetic
ADR	Adverse drug reaction
SSRI	Selective serotonin reuptake inhibitor
SNRI	Serotonin–Norepinephrine Reuptake Inhibitors
CPIC	Clinical Pharmacogenetics Implementation Consortium
DPWG	Dutch Pharmacogenetics Working Group
