# Supplementary material for: Pharmacogenetic Testing of Children and Adolescents with Mental Health Conditions: Real-World Experiences
Source: Pharmaceuticals (Basel). 2025 Aug 8;18(8):1170. doi: 10.3390/ph18081170 (PMC12389199; doi:10.3390/ph18081170)
Supplement: Supplementary file 1 [file pharmaceuticals-18-01170-s001.zip › pharmaceuticals-3753038-supplementary.pdf]

Table S1. Cohort (N=100) use of psychotropic medications that do not have available clinical PGx guidance based on *CYP2C19* or *CYP2D6* genotyping from CPIC or DPWG

Class	Medication	Proportion of Participants (%)
Anxiolytic	Buspirone	1
Atypical antipsychotic	Lurasidone	1
	Olanzapine	8
	Paliperidone	1
	Quetiapine	14
Benzodiazepine	Lorazepam	14
	Nitrazepam	1
Centrally acting alpha2A-adrenergic receptor agonist	Guanfacine	35
Centrally acting alpha-agonist hypotensive agents	Clonidine	17
Central nervous system stimulant	Amphetamine or Dextroamphetamine	19
	Lisdexamphetamine	35
	Methylphenidate	55
Norepinephrine and dopamine reuptake inhibitor	Bupropion	9
Selective serotonin and norepinephrine reuptake inhibitors	Duloxetine	8
Selective serotonin reuptake inhibitor	Fluoxetine	46
Serotonin receptor antagonists and reuptake inhibitors	Trazadone	6
Serotonin–norepinephrine reuptake inhibitor	Desvenlafaxine	6
	Levomilnacipran	1
Tetracyclic antidepressant	Mirtazapine	8
Tricyclic antidepressant	Doxepin	1
	Loxapine	2

Table S2. ***CYP2C19*** **and** ***CYP2D6*** **diplotype frequencies observed in the cohort**

	Diplotype	Phenotype	Frequency in Cohort
CYP2C19	*17/*17	UM	0.03
	*1/*17	RM	0.22
	*1/*1	NM	0.5
	*1/*2	IM	0.13
	*1/*3	IM	0.01
	*2/*17	IM	0.08
	*2/*2	PM	0.03
CYP2D6	*2x2/*4	UM	0.01
	*1x2/*1	UM	0.01
	*2x2/*9	UM	0.01
	*1/*2x2	UM	0.02
	*1/*10 HYB	NM	0.01
	*2 /*2	NM	0.01
	*2/*29	NM	0.01
	*2x2/*4 HYB	NM	0.01
	*1/*17x2	NM	0.01
	*1/*17	NM	0.01
	*1/*9	NM	0.01
	*2/*10	NM	0.02
	*2/*2	NM	0.02
	*2/*41	NM	0.03
	*1/*10	NM	0.03
	*1/*41	NM	0.08
	*1/*1	NM	0.13
	*1/*2	NM	0.16
	*5/*9	IM	0.01
	*2/*3	IM	0.01
	*29/*41	IM	0.01
	*2/*5	IM	0.01
	*15x2/*29	IM	0.01
	*5/*41	IM	0.01

	*4/*10	IM	0.01
	*10/*10	IM	0.01
	*1/*6	IM	0.01
	*1/*5	IM	0.02
	*17/*17	IM	0.02
	*2/*4 HYB	IM	0.03
	*4/*41	IM	0.04
	*1/*4	IM	0.06
	*2/*4	IM	0.09
	*4/*6	PM	0.01
	*4/*5	PM	0.01
	*4/*4-*68 HYB	PM	0.01
	*4/*4	PM	0.03

HYB, Hybrid alleles
UM, Ultrarapid metabolizer
RM, Rapid metabolizer
NM, Normal metabolizer
IM, Intermediate metabolizer
PM, Poor metabolizer

Table S3. Description of reported adverse drug reactions (ADRs) across the cohort

		AA		SNRI	SSRI				
**ADR Category**	**Reported ADR**	**Aripiprazole N=29**	**Risperidone N=27**	**Atomoxetine N=25**	**Citalopram N=2**	**Escitalopram N=18**	**Fluvoxamine N=7**	**Sertraline N=46**	**Vortioxetine N=4**
Endocrine/ Metabolic	Gynecomastia		1 (3.7%)						
	Hyperprolactinemia		1 (3.7%)						
	Prolactinoma and weight gain		1 (3.7%)						
	Weight gain	2 (6.9%)	1 (3.7%)						
Gastrointestinal	Nausea and/or vomiting							1 (2.2%)	1 (25%)
Hepatic	Liver injury	1 (3.4%)							
Nervous System	Aggression and altered mood	1 (3.4%)				1 (5.6%)			
	Aggression, disinhibition, manic episode					1 (5.6%)			
	Aggression and/or irritability	1 (3.4%)	1 (3.7%)	2 (8.0%)				4 (8.7%)	
	Aggression and self- harm					1 (5.6%)			
	Agitation and distress							3 (6.5%)	
	Altered appetite			1 (4.0%)					
	Altered mood	2 (6.9%)		1 (4.0%)		1 (5.6%)			
	Altered mood and anxiety							1 (2.2%)	
	Cognitive decline							1 (2.2%)	
	Dizziness, headache, and jitters	1 (3.4%)						1 (2.2%)	
	Extrapyramidal symptoms	1 (3.4%)							
	Fatigue and/or lethargy		1 (3.7%)	2 (8.0%)				1 (2.2%)	
	Fatigue and memory loss							1 (2.2%)	
	Fatigue and headache	1 (3.4%)	1 (3.7%)						

	Headache							1 (2.2%)	1 (25%)
	Hearing voices							1 (2.2%)	
	Hypothermia	1 (3.4%)							
	Increased stimulation		1 (3.7%)						
	Increased suicidal ideation or suicidal ideation	1 (3.4%)		2 (8.0%)				2 (4.3%)	
	Irritability, trouble sleeping		1 (3.7%)						
	Manic reaction/ mania							2 (4.3%)	
	Sedation						1 (14.3%)		
	Uncharacterized behavioural effects		1 (3.7%)					2 (4.3%)	
	Violent outbursts, mood swings	1 (3.4%)							
Oral	Mouth ulcers				1 (50%)			1 (2.2%)	
Skeletal/ Muscular	Rhabdomyolysis		1 (3.7%)						
Skin	Picking at skin					1 (5.6%)			
Combinatorial	Abdominal pain and dizziness					1 (5.6%)			
	Acid reflux/nausea and pruritus/rash							1 (2.2%)	
	Aggression and insomnia							1 (2.2%)	
	Altered behaviours and movements		1 (3.7%)						
	Altered movements and vocalizations							1 (2.2%)	
	Binge eating and weight gain	1 (4.8%)							
	Chest pain, headache, hunger, and increased salivation							1 (2.2%)	
	Decreased appetite and increased tics			1 (4.0%)					

	Erythematous rash and irritability		1 (3.7%)						
	Fatigue and increased appetite		1 (3.7%)						
	Fatigue and nightmares					1 (5.6%)			
	Increased tics and teeth grinding							1 (2.2%)	
	Lethargy and vomiting		1 (3.7%)						
	Lightheadedness, restlessness, and tachycardia							1 (2.2%)	

N, total number of participants who had undergone therapy with the respective psychotropic agent
AA, Atypical antipsychotic
SNRI, Serotonin and norepinephrine reuptake inhibitor
SSRI, Selective serotonin reuptake inhibitor

Table S4. Interpretation of Clinical PGx Guidelines

Medication	Treatment Response Considered to be Consistent with Metabolizer Status	Metabolizer Status(es)	Reference
**Amitriptyline**	ADR	CYP2C19 PM + CYP2D6 UM<br>CYP2C19 PM + CYP2D6 NM<br>CYP2C19 PM + CYP2D6 IM<br>CYP2C19 PM + CYP2D6 PM<br>CYP2C19 IM + CYP2D6 IM<br>CYP2C19 IM + CYP2D6 PM<br>CYP2C19 NM + CYP2D6 IM<br>CYP2C19 PM + CYP2D6 PM<br>CYP2C19 RM + CYP2D6 IM<br>CYP2C19 RM + CYP2D6 PM<br>CYP2C19 RM + CYP2D6 NM<br>CYP2C19 RM + CYP2D6 UM<br>CYP2C19 UM + CYP2D6 IM<br>CYP2C19 UM + CYP2D6 PM<br>CYP2C19 UM + CYP2D6 NM<br>CYP2C19 UM + CYP2D6 UM	[1]
	Inefficacy	CYP2C19 UM + CYP2D6 UM<br>CYP2C19 UM + CYP2D6 NM<br>CYP2C19 UM + CYP2D6 IM<br>CYP2C19 UM + CYP2D6 PM<br>CYP2C19 RM + CYP2D6 UM<br>CYP2C19 RM + CYP2D6 NM<br>CYP2C19 RM + CYP2D6 IM<br>CYP2C19 RM + CYP2D6 PM<br>CYP2C19 NM + CYP2D6 UM<br>CYP2C19 IM + CYP2D6 UM<br>CYP2C19 PM + CYP2D6 UM	[1]
**Aripiprazole**	ADR	CYP2D6 PM	[2]
	Inefficacy	N/A	[2]
**Atomoxetine**	ADR	CYP2D6 PM	[3]
	Inefficacy	CYP2D6 UM	[3]

**Brexpiprazole**	ADR	CYP2D6 PM	[2]
	Inefficacy	N/A	[2]
**Citalopram**	ADR	CYP2C19 IM<br>CYP2C19 PM	[4]
	Inefficacy	CYP2C19 UM<br>CYP2C19 RM	[4]
**Clomipramine**	ADR	CYP2C19 UM + CYP2D6 UM<br>CYP2C19 UM + CYP2D6 NM<br>CYP2C19 UM + CYP2D6 IM<br>CYP2C19 UM + CYP2D6 PM<br>CYP2C19 RM + CYP2D6 UM<br>CYP2C19 RM + CYP2D6 NM<br>CYP2C19 RM + CYP2D6 IM<br>CYP2C19 RM + CYP2D6 PM<br>CYP2C19 PM + CYP2D6 UM<br>CYP2C19 PM + CYP2D6 NM<br>CYP2C19 PM + CYP2D6 IM<br>CYP2C19 PM + CYP2D6 PM<br>CYP2C19 IM + CYP2D6 IM<br>CYP2C19 NM + CYP2D6 IM<br>CYP2C19 IM + CYP2D6 PM<br>CYP2C19 NM + CYP2D6 PM	[1]
	Inefficacy	CYP2C19 UM + CYP2D6 UM<br>CYP2C19 UM + CYP2D6 NM<br>CYP2C19 UM + CYP2D6 IM<br>CYP2C19 UM + CYP2D6 PM<br>CYP2C19 RM + CYP2D6 UM<br>CYP2C19 RM + CYP2D6 NM<br>CYP2C19 RM + CYP2D6 IM<br>CYP2C19 RM + CYP2D6 PM<br>CYP2C19 PM + CYP2D6 UM<br>CYP2C19 PM + CYP2D6 NM<br>CYP2C19 PM + CYP2D6 IM<br>CYP2C19 PM + CYP2D6 PM<br>CYP2C19 IM + CYP2D6 UM<br>CYP2C19 NM + CYP2D6 UM	[1]
**Escitalopram**	ADR	CYP2C19 IM	[4]

		CYP2C19 PM	
	Inefficacy	CYP2C19 UM<br>CYP2C19 RM	[4]
**Fluvoxamine**	ADR	CYP2D6 IM<br>CYP2D6 PM	[4]
	Inefficacy	N/A	[4]
**Risperidone**	ADR	N/A	[2]
	Inefficacy	CYP2D6 UM	[2]
**Sertraline**	ADR	CYP2C19 PM	[4]
	Inefficacy	N/A	[4]
**Venlafaxine**	ADR	CYP2D6 PM	[4]
	Inefficacy	N/A	[4]
**Vortioxetine**	ADR	CYP2D6 IM<br>CYP2D6 PM	[4]
	Inefficacy	CYP2D6 UM	[4]

PGx, pharmacogenetic
ADR, adverse drug reaction
NA, not applicable
UM, ultrarapid metabolizer
RM, rapid metabolizer
NM, normal metabolizer
IM, intermediate metabolizer
PM, poor metabolizer

References

1. Hicks JK, Sangkuhl K, Swen JJ, et al. Clinical pharmacogenetics implementation consortium guideline (CPIC) for CYP2D6 and CYP2C19 genotypes and dosing of tricyclic antidepressants: 2016 update. *Clin Pharmacol Ther*. 2017;102(1):37-44. doi:10.1002/cpt.597
2. Beunk L, Nijenhuis M, Soree B, et al. Dutch Pharmacogenetics Working Group (DPWG) guideline for the gene-drug interaction between CYP2D6, CYP3A4 and CYP1A2 and antipsychotics. *Eur J Hum Genet*. 2024;32(3):278-285. doi:10.1038/s41431-023-01347-3
3. Brown JT, Bishop JR, Sangkuhl K, et al. Clinical Pharmacogenetics Implementation Consortium Guideline for Cytochrome P450 (CYP)2D6 Genotype and Atomoxetine Therapy. *Clin Pharmacol Ther*. 2019;106(1):94-102. doi:10.1002/cpt.1409
4. Bousman CA, Stevenson JM, Ramsey LB, et al. Clinical Pharmacogenetics Implementation Consortium (CPIC) Guideline for CYP2D6, CYP2C19, CYP2B6, SLC6A4, and HTR2A Genotypes and Serotonin Reuptake Inhibitor Antidepressants. *Clin Pharmacol Ther*. 2023;114(1):51-68. doi:10.1002/cpt.2903
